# Supplementary material for: Passive muscle forces in Drosophila are large but insufficient to support a fly’s weight
Source: bioRxiv. 2025 Aug 2:2025.04.29.651225. Preprint. [Version 2] doi: 10.1101/2025.04.29.651225 (PMC12324252; doi:10.1101/2025.04.29.651225)
Supplement: Supplement 3 [file NIHPP2025.04.29.651225v2-supplement-3.pdf]

bioRxiv preprint doi: https://doi.org/10.1101/2025.04.29.651225; this version posted August 2, 2025. The copyright holder for this preprint (which was not certified by peer review) is the author/funder, who has granted bioRxiv a license to display the preprint in perpetuity. It is made available under aCC-BY 4.0 International license.

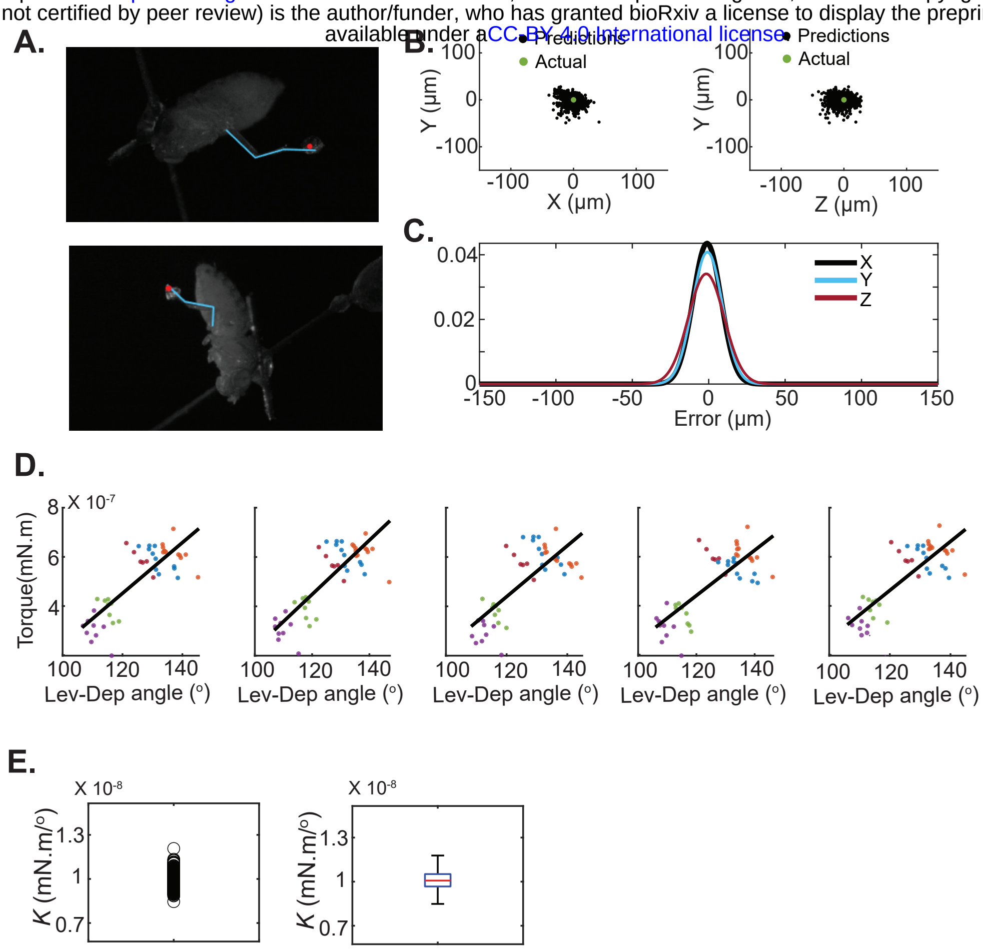

Figure 2-S1. Reconstruction errors resulting from the camera calibration are small.
A. To test the accuracy of calibration coefficients obtained from the DLT camera calibration, we used inverse DLT to obtain pixel coordinates from the 3D-reconstructed XYZ leg joint positions and overlaid these points on the corresponding frames. The reconstructed leg segments lie on top of the actual leg.
B. Error distribution across each axis for DLT camera calibrations used for 3D reconstruction of leg kinematics. Error values were obtained by 100 iterations of random subsampling of control points into two sets: The training set for calculating calibration coefficients and the testing set for evaluating prediction accuracy.
C. The error distribution for each camera calibration is well-captured by a normal distribution.
D. We sampled from the Gaussian distribution in C to create 1000 simulated legs. This figure shows 4 randomly selected simulations. The first panel shows the original data, while the other four show randomly selected iterations. The variation in results due to DLT error propagation is small.
E. Final stiffness distributions for levation-depression from all 1000 iterations of DLT error propagation simulations show that variation in results due to DLT error is small.

bioRxiv preprint doi: https://doi.org/10.1101/2025.04.29.651225; this version posted August 2, 2025. The copyright holder for this preprint (which was not certified by peer review) is the author/funder, who has granted bioRxiv a license to display the preprint in perpetuity. It is made available under aCC-BY 4.0 International license.

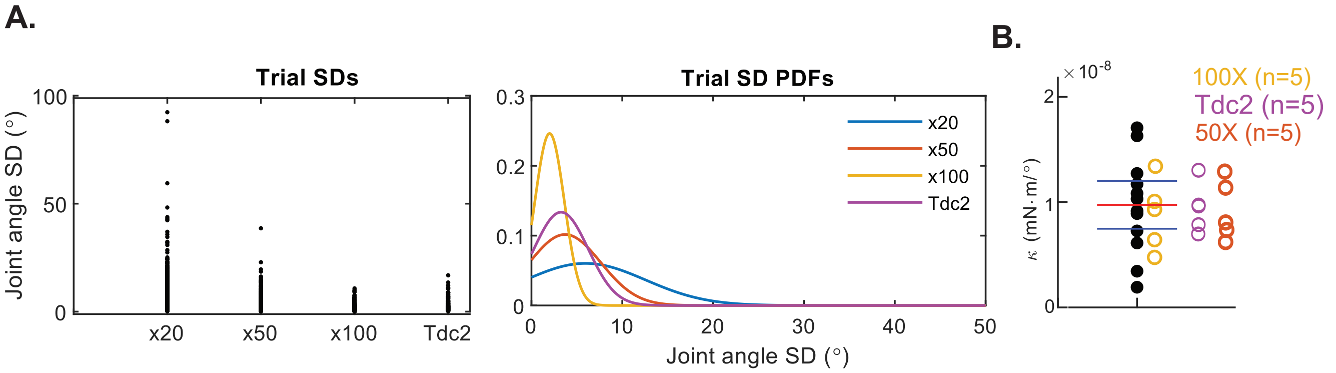

Figure 2-S2. Larger weight and silencing octopaminergic neurons does not affect spring constant measurements. A. Standard deviations (SDs) of joint angle values across experimental conditions: x20 etc. refers to the mass of the additional weight. x20, x50, x100 means 20,50,100 times the mass of the leg, respectively. Three masses were used. Tdc2 refers to the case when both glutamatergic and octopaminergic neurons are silenced. Tdc2 experiments were performed with x50 weight. Joint angle SD values per trial for each experimental condition. Probability density functions for trial joint angle SDs by experimental condition. Higher weight reduces the standard deviation. There is no further decrease in SD when both glutamatergic and octopaminergic neurons are silenced.
B. Stiffness values obtained from experiments with higher weights and when octopaminergic neurons are inactivated. The values lie within the interquartile range observed with the smaller weights.

Octopaminergic neuromodulation is silenced optogenetically along with motor neurons using the genetic construct Tdc2-Gal4;UAS-GtACR1(III) x VGlut(OK371)-Gal4(II). For all other flies, only motor neurons are optogenetically silenced via the construct VGlut(OK371)-Gal4(II) x UAS-GtACR1(III).

bioRxiv preprint doi: https://doi.org/10.1101/2025.04.29.651225; this version posted August 2, 2025. The copyright holder for this preprint (which was not certified by peer review) is the author/funder, who has granted bioRxiv a license to display the preprint in perpetuity. It is made available under aCC-BY 4.0 International license.

A.

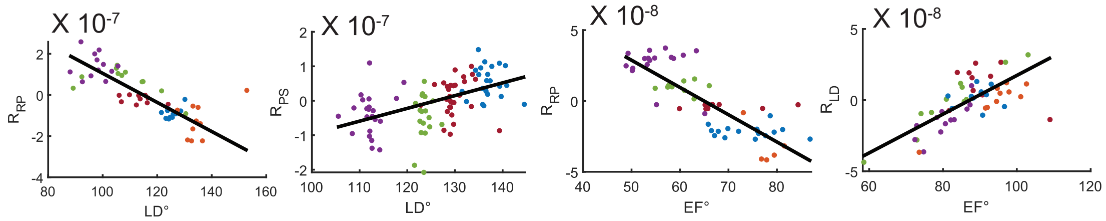

B.

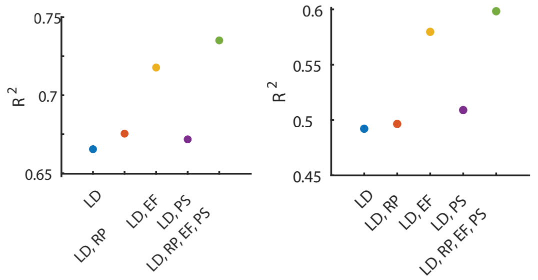

Figure 2 S3. Variation in torque can be explained by the coupled actions of multiple degrees of freedom, as well as other internal forces.

A. Examples showing that the angle at one degree of freedom is correlated with residuals (torque that is not explained by changes at the original angle) in other degrees of freedom suggesting that torque variation at one degree of freedom (DOF) may depend on other DOFs. As an explicit example of this analysis, for the leftmost panel in A, we first fit the retraction-protraction torque to the changes in retraction-protraction angle as shown in Figures 2-3. Residuals are calculated as the difference between the actual torque and linear fit and are plotted here against the value of the levation-depression angle on the same torque. A significant correlation is found in >60% of cases implying a coupling between the different angles. Importantly, these correlations are likely to be second-order effects.

B. Using multiple DOF angles to predict levation-depression (LD) torque results in a higher R-squared value than when using only the LD angle. We did not perform this analysis systematically. However, in the 6 flies (just to test the idea), we performed the analysis on we found that there was a much higher correlation when other degrees of freedom were used. Extension-flexion degrees of freedom seem to have a particularly large effect. Levation-depression for two of the flies are shown.
